# Application of Intraoperative Ultrasound Navigation in Neurosurgery

**DOI:** 10.3389/fsurg.2022.900986

**Published:** 2022-05-10

**Authors:** Keith Simfukwe, Iurii Iakimov, Rinat Sufianov, Luís Borba, Luciano Mastronardi, Alina Shumadalova

**Affiliations:** ^1^Federal Center of Neurosurgery, Tyumen, Russia; ^2^Department of Neurosurgery, Sechenov First Moscow State Medical University (Sechenov University), Moscow, Russia; ^3^Department of Neurosurgery, Federal University of Paraná, Curitiba, Brazil; ^4^Division of Neurosurgery, San Filippo Neri Hospital, Roma, Italy; ^5^Depatment of General Chemistry, Bashkir State Medical University, Ufa, Russia

**Keywords:** ultrasonography, navigation, neurosurgery, epilepsy, glioma

## Abstract

Effective intraoperative image navigation techniques are necessary in modern neurosurgery. In the last decade, intraoperative ultrasonography (iUS), a relatively inexpensive procedure, has gained widespread acceptance.

**Aim:**

To document and describe the neurosurgery cases, in which iUS has been employed as the primary navigational tool. This includes a discussion of the advantages that iUS may possess relative to other forms of neuronavigation.

**Conclusion:**

The application of iUS as an intraoperative navigation tool during neurosurgery holds great potential as it has been shown, relative to other neuronavigation techniques, to be quick, repeatable, and able to provide real-time results.

## Introduction

The ideal intraoperative navigational modality for neurosurgeons is the one that is accurate, user-friendly, and most of all cost-efficient (1–4). Over the last two decades, the most reliable and frequently used intraoperative navigational tools include intraoperative computed tomography (iCT) and intraoperative magnetic resonance imaging (iMRI), with the latter being deemed the “golden standard” (5–7). Despite the effectiveness of the aforesaid intraoperative tools, neurosurgeons still face challenges in the acquisition and use of iCT and iMRI. These challenges include the following: (1) Enormous costs in the acquisition of these tools, especially in low-budget neurosurgical centers and developing countries; (2) Heavy dependence on acquired pre-operative images; (3) At every stage of the procedure; post craniotomy, dural opening, tumor debulking, and resection, the element of brain shift results in varying degrees of loss of accuracy; (4) Inherent lack of soft-tissue resolution and the associated radiation exposure like in the case of iCT. To circumvent these hurdles, more innovative, convenient, and novel generation modes of Intraoperative sonography or ultrasound (iUS) systems have been developed. The iUS is comparatively inexpensive, easy to use, and requires less intraoperative preparation. The two-dimensional (2D) greyscale and three-dimensional (3D) iUS provide real-time, clear, and well-correlated images, which are easily interpreted by the neurosurgeon. Additionally, the ease and flexibility it provides the user make it possible to counter-check the location of the lesion at any stage of the surgery without prolonged workflow stoppages (8). This, therefore, solves the element of brain shift. The iUS does not require pre-existing images before surgery. However, there is a necessity to save the initial image scanned to serve as a baseline check during the surgery. In this narrative, we reclaiming these findings by outlining our center (Federal Center of Neurosurgery, Tyumen, Russia) experience in the use of iUS during brain, spine, vascular, and epilepsy surgery. We do this by highlighting technical nuances and discussing, with illustrative cases, the spectrum of applications and benefits. Finally, we descriptively evaluate neurosurgeons' experience in using iUS as a neuronavigation tool.

## Methodology

We took a retrospective approach for this investigation, and it involved the extraction of data from a facility database on neurosurgical procedures that were performed between 2015 and December of 2021 at the Federal Center of Neurosurgery, Tyumen Russia. The key criterion for inclusion in the investigation was the documented intraoperative application of iUS during elective surgery. The investigators reviewed a total of 1,330 patient records, with documented brain and spinal lesions. In addition, we also assessed neurosurgeon's experience with the use of intraoperative ultrasound as a neuroimaging through the application of an online survey tool the following link: https://docs.google.com/forms/d/e/1FAIpQLSfqHcExcft3FwcTKiKb2A0b5i828UhnoXe1smoXPDIdH6_g/viewform?usp= sf_link (Table 1).

**Table 1:** Survey on the applicability of IUS in neurosurgery as a neuronavigation tool.

**Participants (41) Country (15)**	**Russia (20), Spain (5), Zambia (3), India (2), Algeria (1), Costarica (1), France (1), Greece (1), Iraq (1), Kyrgystan (1), Nepal (1), Uzbekistan (1), Ethiopia (1), Mongolia (1), Palestine (1)**																
**Questions**	**Yes**	**No**	**ST**	**N**	**IDK**	**G**	**B**	**VT**	**NTL**	**F**	**X**	**Ius**	**CT**	**Mri**	**N**	**Fmri**	**Non**
Q1. Familiar with neuro-Imaging tools										41.5	68.3	73.	53.7	43.9	78	43.9	2.4
Q2. Neuron Imaging tools are available in facilities										56.1	80.5	78	53.7	41.7	78	53	2.4
Q3. Do you use Intraoperative neuroimaging during brain tumor surgery	51.2	17.1	31.7							
Q4. Have you experienced brain shift during tumor brain surgery^*^	41.5	19.5	26.8	2.4	9.8					
Q5. Are you familiar with ultrasound intraoperative neuroimaging during brain tumor surgery?	81.8	19.2								
Q6. Dose the use of intraoperative ultra sound neuro imaging improve tumor resection outcome?	73.2	4.9	22							
Q7. How would you grade the use of intraoperative ultrasound neuoimaging during brain tumor surgery?						90.3	9.7			
Q8. How long dose did it take you to learn and apply usage of intraoperative ultrasound?								58.5	41.5	
Q9. How long dose it take to apply usage of intraoperative ultrasound during a procedure?								29.2	70.8	
Epilepsy surgery		GTR	STR	B	NA
Q10. Do you manage patients with intractable epilepsy?		34.1	17.1							
Q11. Do you use intraoperative neuroimaging during epilepsy surgery?	48.8	39	17.1							
Q12. Have you used intraoperative ultrasound neuroimaging during epilepsy surgery?	43.9	53.7	14.6							
Q13. How would you grade the EOR with aid of intraoperative ultrasound neuroimaging during epilepsy surgery?	31.7									53.6	4.9	4.9	36.6

*St, Sometimes; N, never; IDK, I don't Know; G-Good; B, Bad; NVT, long time; VT, very long time; F, fluoroscopy ; X, X-ray; IUS, intra operative ultrasound; CT, computer temography; EOR, extent of resection; GTR, Gross total resection; STR, subtotal resection; B, biopsy; NA, not attempted*.

* *Brain shift is the change of a brain lesion position from it's original anatomically located point on MRI. All results are in percentages*.

### Equipment and Technical Modality

**Figure 1:**
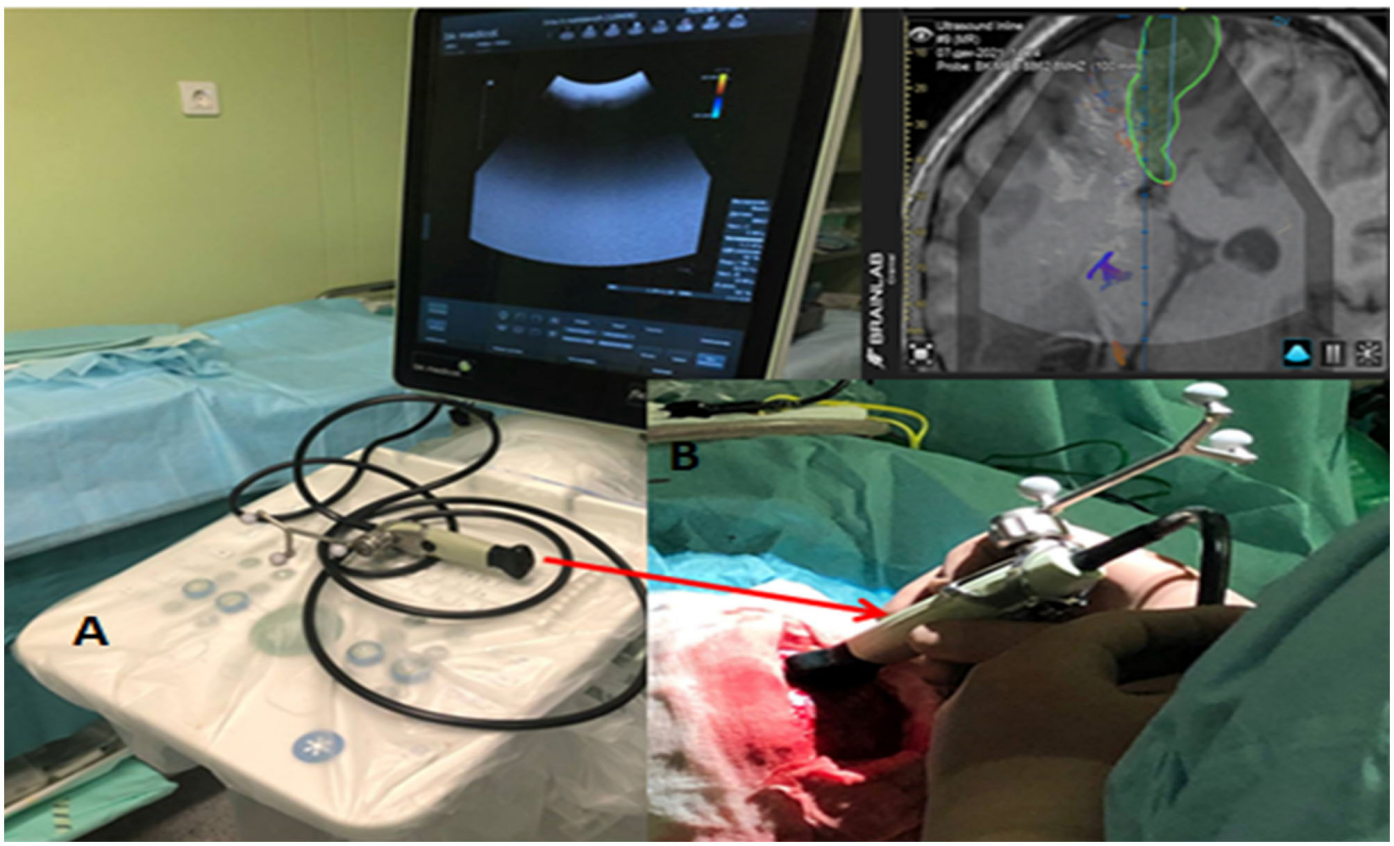
**(A)** iUS Apparatus - BK Medical flex focus 800 (Denmark) with Sterile convex transducer. **(B)** Integrated Brain lab neuronavigation system (Germany).

We employed a FlexFocus 800 iUS [BK Medical, Denmark for three-dimensional (3D)] iUS neuro-navigation. We applied a Linear-type and convex transducer (Figure 1B; high-frequency Linear 8870), as well as a craniotomy sensor (Craniotomy 8862). We use a frequency of 3.8–10 MHz, a contact surface of 29 × 10 mm. The iUS was done in coronal, sagittal, and axial planes.

**Figure 2:**
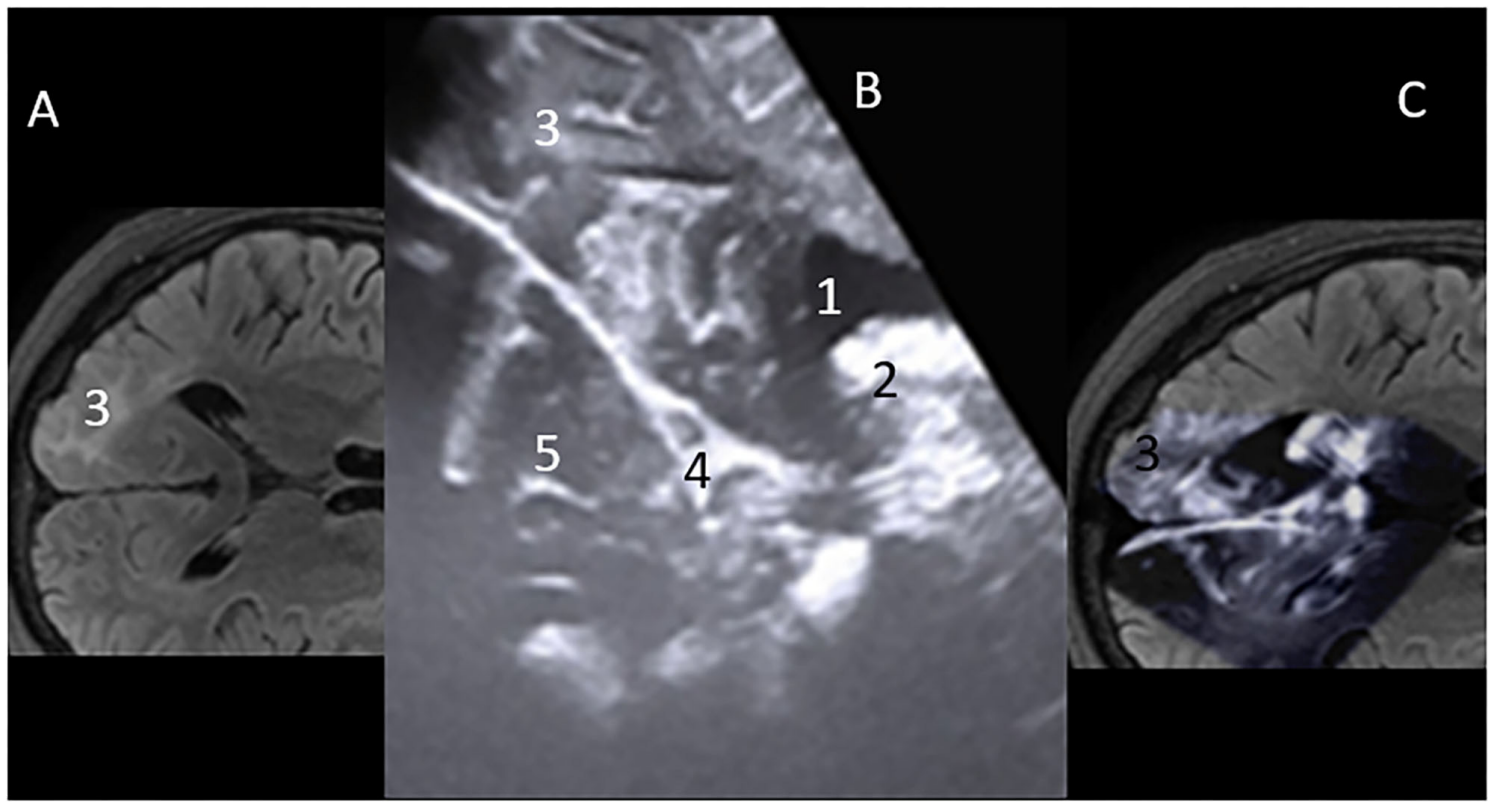
**(A)** MRI – flair axial view of Rt occipital lobe Focal cortical dysplasia (FCD) lesion-3. **(B)** Mirror iUS Axial view of Rt occipital lobe FCD lesion-3. Cerebral spinal fluid (CSF) in ventricle occipital horn is “Hypoechoic” on iUS-1; “Hyperechoic” futures vascular stuctures, high grade tumors. Here, the choroid plexis is hyperechoic-2. Falx- 4. Normal brain tissue on iUS is “isoechoic”-5. **(C)** Projection on the intraoperative Ultrasound Concordance of lesion location on MRI and iUS.

Anatomically consistent reference points, as mentioned above, were used for the localization of the lesions. The interpretation of anatomical features on iUS was described by their respective echogenicity reflections' tissue consistency. We described spaces that had no echogenicity, such as ventricles/ CSF spaces as anechogenic. Hypoechoic features were defined in spaces with less echogenicity, such as the brainstem. Isoechogenic structures included the normal brain tissue (white matter) and conversely hyperechoic in tissues with high enchogenicity, such as in gliomas, calcifications, meningiomas, and vascular anatomical structures, such as the choroid plexus (Figure 2). Imaging characteristics on iUS were compared with pre- and post-operative CT or MRI images available to assess for concordance.

## Results

### Survey on the Applicability of iUS in Neurosurgery as a Neuronavigation Tool

There are 41 neurosurgeons, 33 males, and eight females, from 15 countries, 12 of whom were from low-and-middle-income countries (LMIC), while three were from middle-income (MIC) and high-income countries (HIC). Regarding years of experience, 73.8% had, at most, 5 years of working experience as neurosurgeons, being relatively junior residents, while 26.2% had at least 6 years of working experience, being individuals at the registrar to consultant professor level.

### iUS-Navigated Neurosurgical Cases

All the surgeries were performed by Professor Albert Sufianov at the Federal Center of Neurosurgery, Tyumen, Russia, with the assistance of the same surgical team. Intraoperative ultrasonography was performed after the craniotomy, both before and after opening the dura mater.

### Supratentorial Low-Grade Glioma

**Figure 3:**
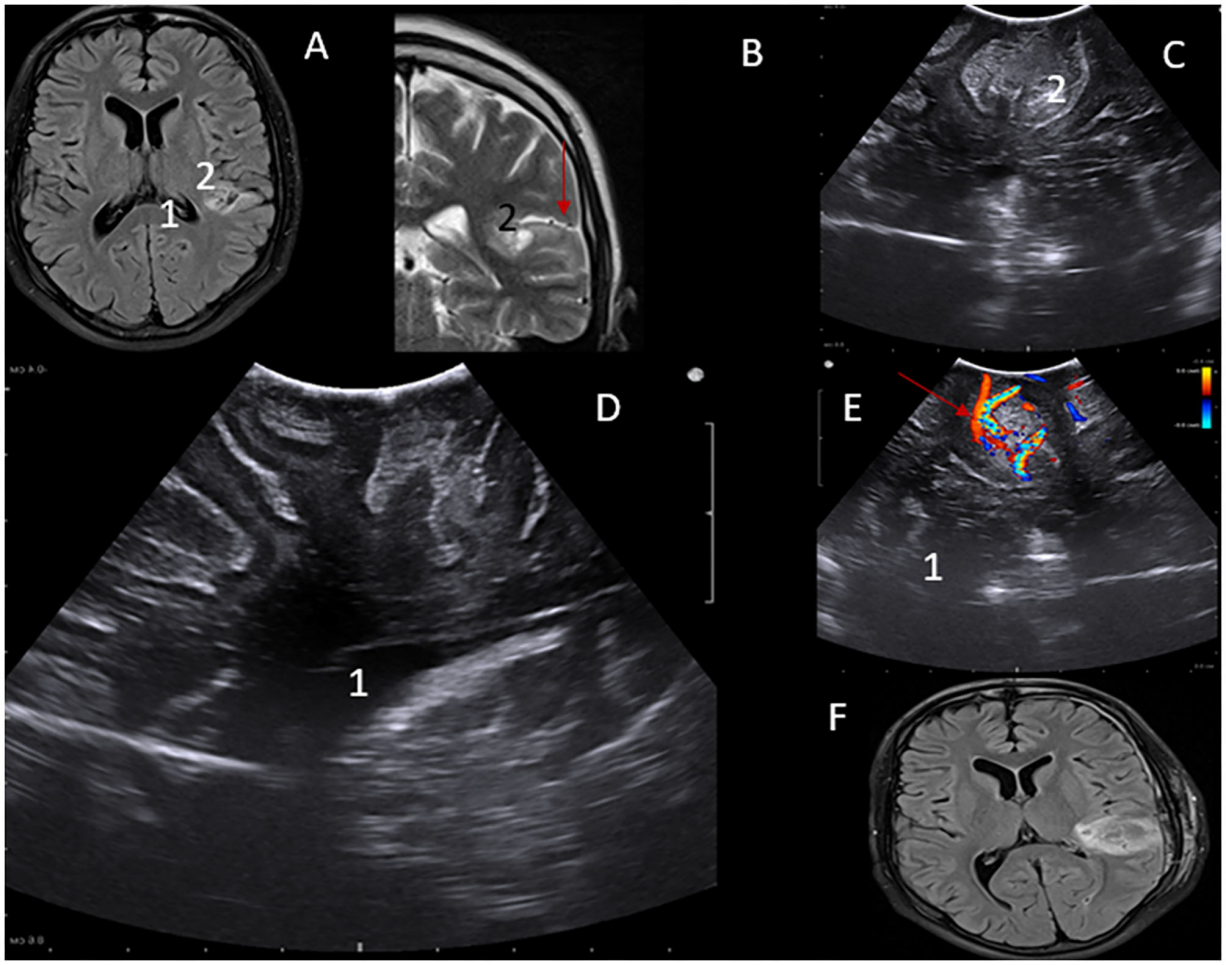
**(A)** MRI – flair axial view of Lt parital lobe lesion- 1. **(B)** MRI – T2WI MRI coronal view of Lt parietal lobe lesion- 2. iUS depiction of low-grade lesion in axial. **(C,D)** Saggital views Perilesional vascular structures can be visualized using Color Doppler function-Red arrow **(A,E)**. Post-operative MRI showing gross total resection of the lesion **(F)**.

Low-grade subcortical gliomas can be difficult to locate after the dura mater has been opened. However, as shown in Figure 3, LGG are readily identified, and their margins are well-defined by intraoperative ultrasound regardless of pre-operative imaging patterns. This enhances intraoperative lesion delineation and the extent of resection. Features of lesion-vascular interactions and landscape can be also demonstrated while localizing lesions, a vital component for undesirable hemorrhage avoidance.

### Deep High-Grade Gliomas

**Figure 4:**
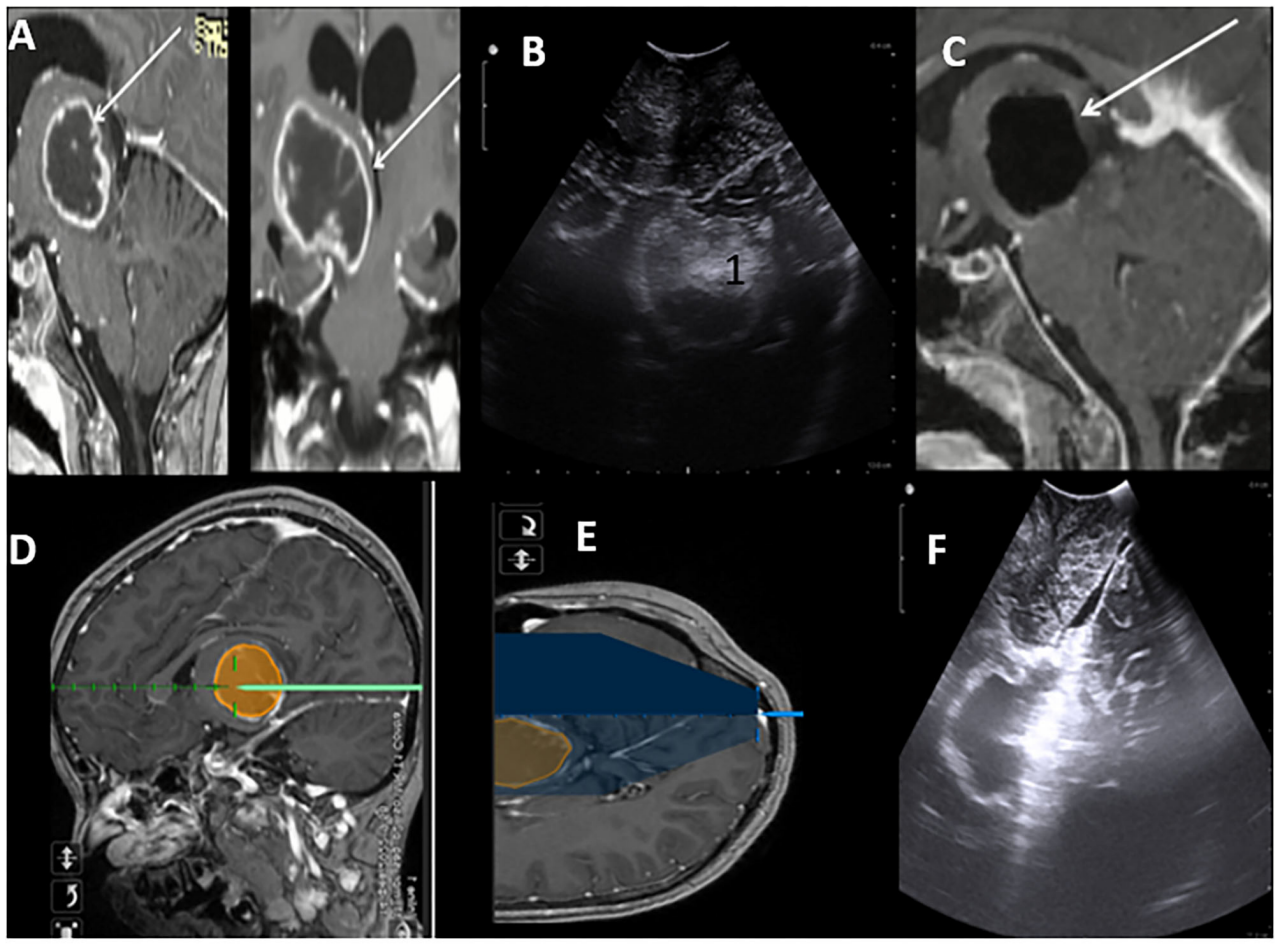
Post Contrast MRI T1WI Sagittal and Coronal sections suggestive of high-grade thalamic glioma (HGG) with cystic central cystic component **(A)**. Brain lab navigation system depicting trajectory to the lesion **(D,E)**. iUS Axil section depicting thalamic hyperechoic lesion **(B)**. Post-resection iUS Sagital section depicting wall of the cavity after complete resection **(F)**. MRI in concordance with ius showing gross total resection of the HGG **(C)**.

One of the key challenges during the surgical treatment of high-grade gliomas is the achievement of the optimum balance between maximizing the resection of the lesion and minimizing the effect on healthy tissue (and any potential neurological impairment). It is in this regard that iUS demonstrates one of its key strengths in being able to provide real-time information on the lesion's location and boundaries. On iUS, they demonstrate varying levels of echoicity and are normally heterogenic. Despite being able to detect deep HGGs, intraoperative evaluation of the extent of resection of deep high-grade gliomas may be challenging when in B-mode iUS. This is because both malignant tumor tissue and peritumoral edema are hyperechoic (Figure 4F). This poses a notable disadvantage compared to iMRI. It is for this reason that we still advocate for early post-operative MRI images and clinical (at 3 and 6 months, then once a year) follow-up to rule out a residual tumor.

### Intraventricular Lesions

**Figure 5:**
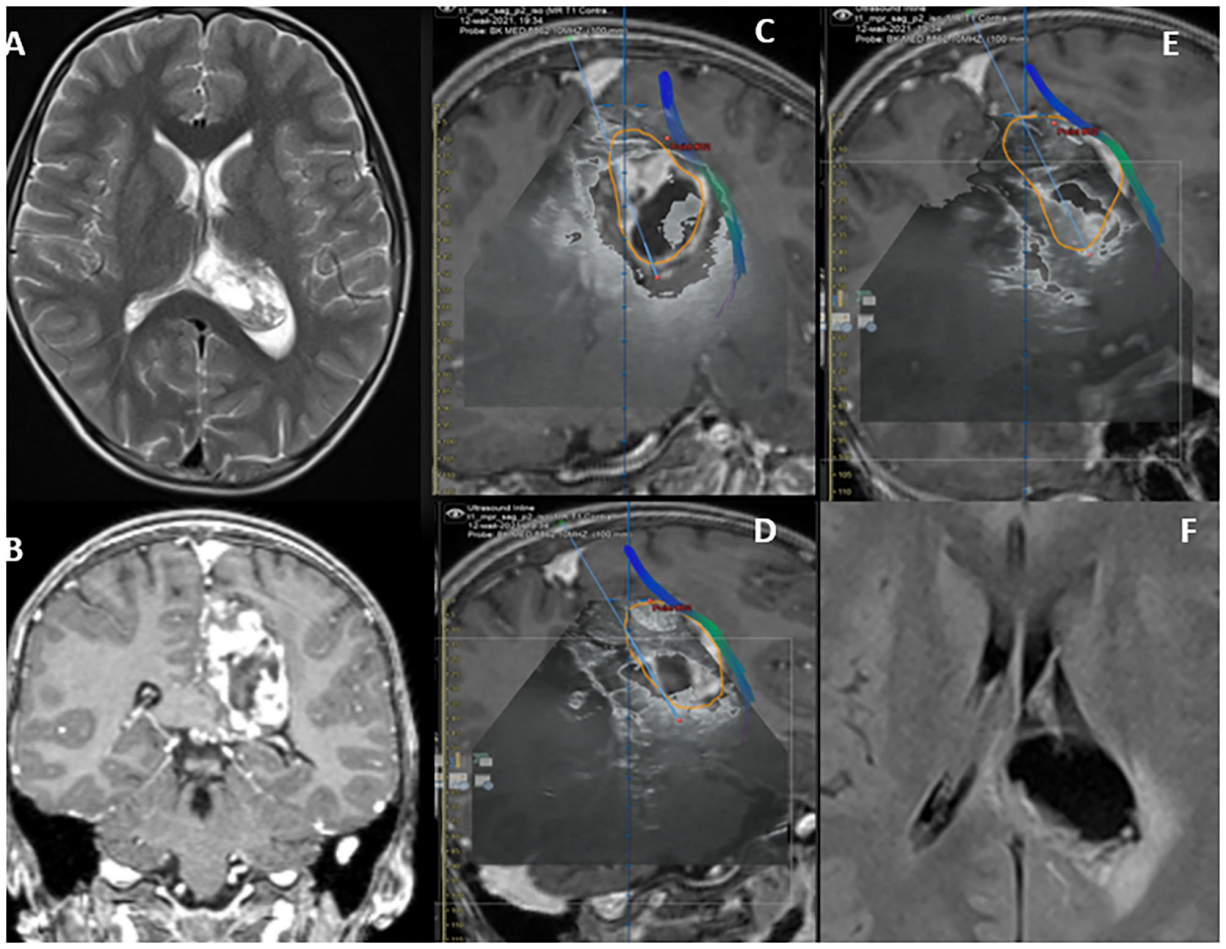
MRI T2 WI Axial parietal- intraventricular cystic lesion **(A)**. Heterogeneous enhancing on T1WI-C as depicted on its coronal section **(B)**. The lesion boundaries are depicted in orange on **(C–E)**, illustrating “Brain shift.” Lesion in close proximity with the cortical spinal tracts. Plain axial post-operative TIWI depicting complete resection of the lesion.

**Figure 6:**
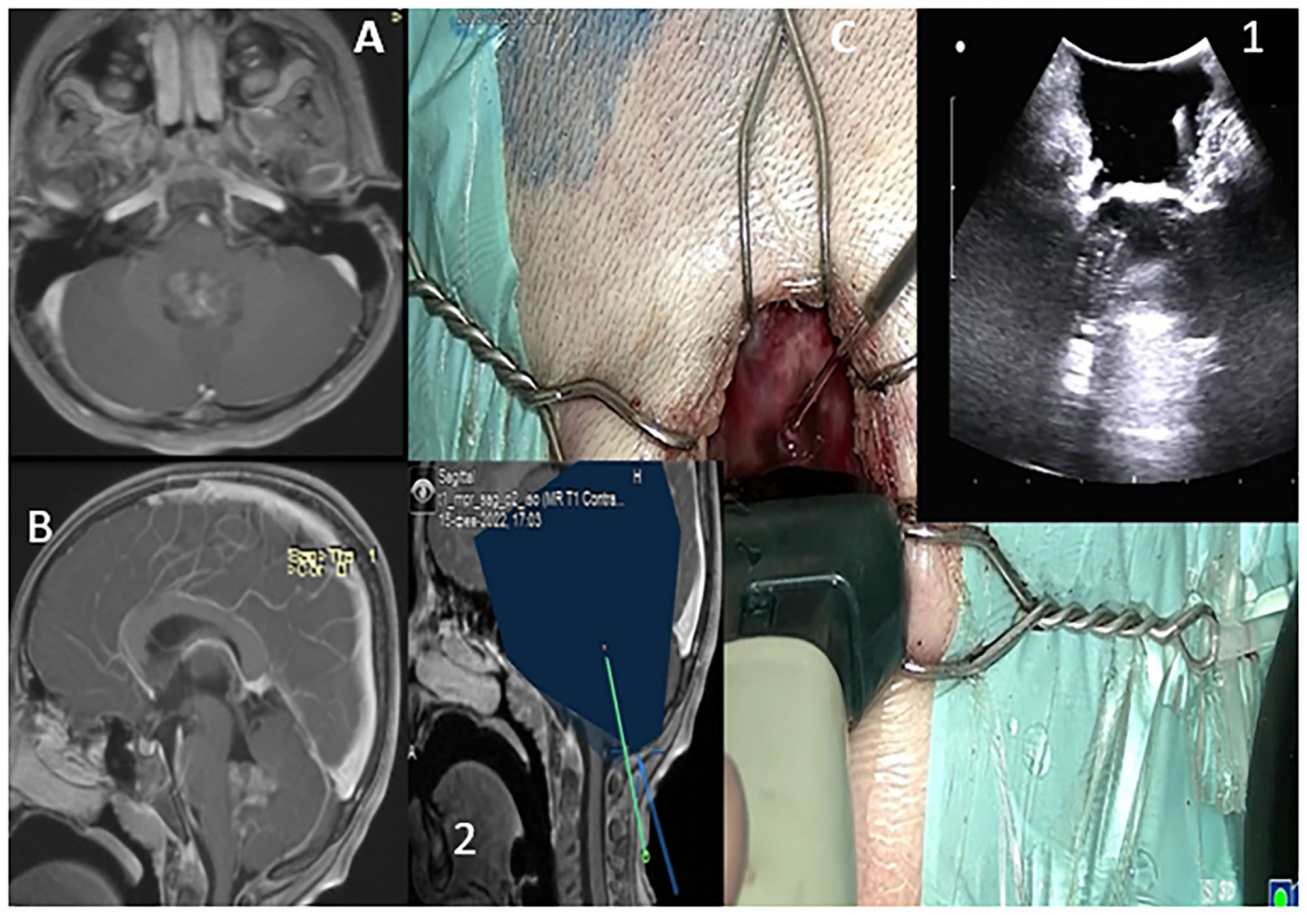
T1WI in sagittal and axial sections depicting 4th ventricular lesion **(A,B)**. Probe is placed over the dura to acquire standard B-mode imaging scans *via* a sub occipital approach. The lesion is depicted as hyperechoic incaved between cerebellar tonsils **(C,1)**. The trajectory to the lesion is also depicted **(C,2)**.

**Figure 7:**
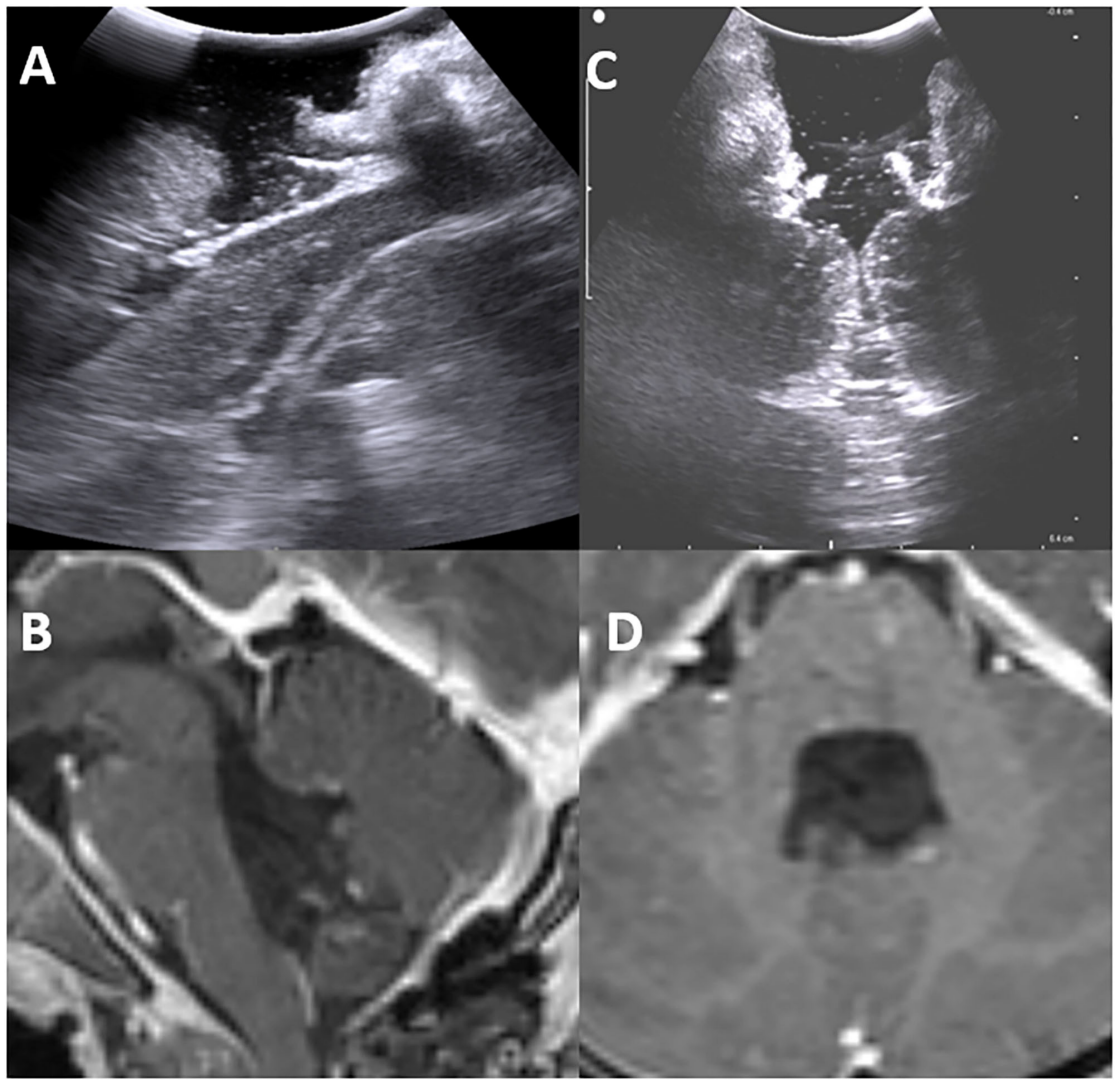
Post-operative intraoperative ultrasound images in sagittal and axial view showing total resection of the lesion **(A,C)** in concordance with postoperative MRI -TIWI in sagittal and axial views **(B,D)**.

In Figures 5, 6, we illustrate two cases of lateral and fourth ventricular lesions resected with iUS guidance along with Bain lab neuronavigation. As illustrated, iUS can be used to highlight even deep-seated brain lesions, to show their relationships with surrounding neural and vascular structures, and to provide real-time and dynamic imaging. As in the previously described cases, the probe is placed over the dura to acquire standard B-mode imaging scans. The lesion is identified on the two axes and is measured. The iUS with brain lab integration, gives greater details of the dimensions of the lesion, about the normal cortical structure during each stage of the procedure, and, therefore, abetting the element of brain shift (Figures 5C–E). Even within narrow and deep corridors to the fourth ventricle, lesion dimensions can be captured on the coronal and sagittal axis. Intricate lesion-anatomical structure interrelations of the fourth ventricle can be clearly defined (Figures 6, 7).

### Vascular Lesions

#### Arteriovenous Malformation

**Figure 8:**
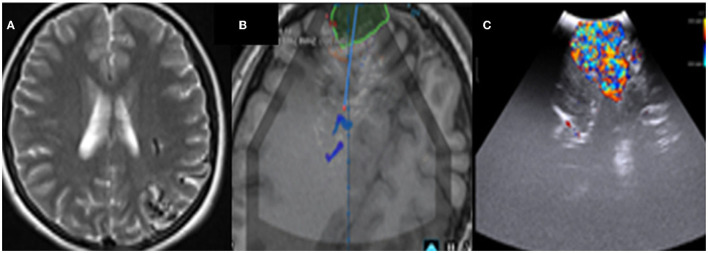
**(A)** Pre-operative MRI depicting an occipital arteriovenous-malformation (AVM). **(B)** Intraoperative ultrasound integrated with brain lab system clearly identifying the AVM. **(C)** Doppler mode.

The iUS can be used to visualize a range of vascular abnormalities, including arteriovenous malformations (AVM). We illustrate our experience in a case of a 17-year-old female who presented with seizures and worsening headaches. Preliminary CT and MRI were suggestive of a 4-cm left occipital AVM (Spetzer Martin Grade 3). The iUS's Doppler mode allows for the precise imaging of arterial feeders and venous drainage. This mode is extremely valuable in AVM surgery because it depicts vascular flow in real-time. Locating the main arteries and veins of a tumor, as well as the patency of the venous sinuses and proof of the tumor's vascularity, aids in safe removal (Figure 8).

### Spinal Lesions

**Figure 9:**
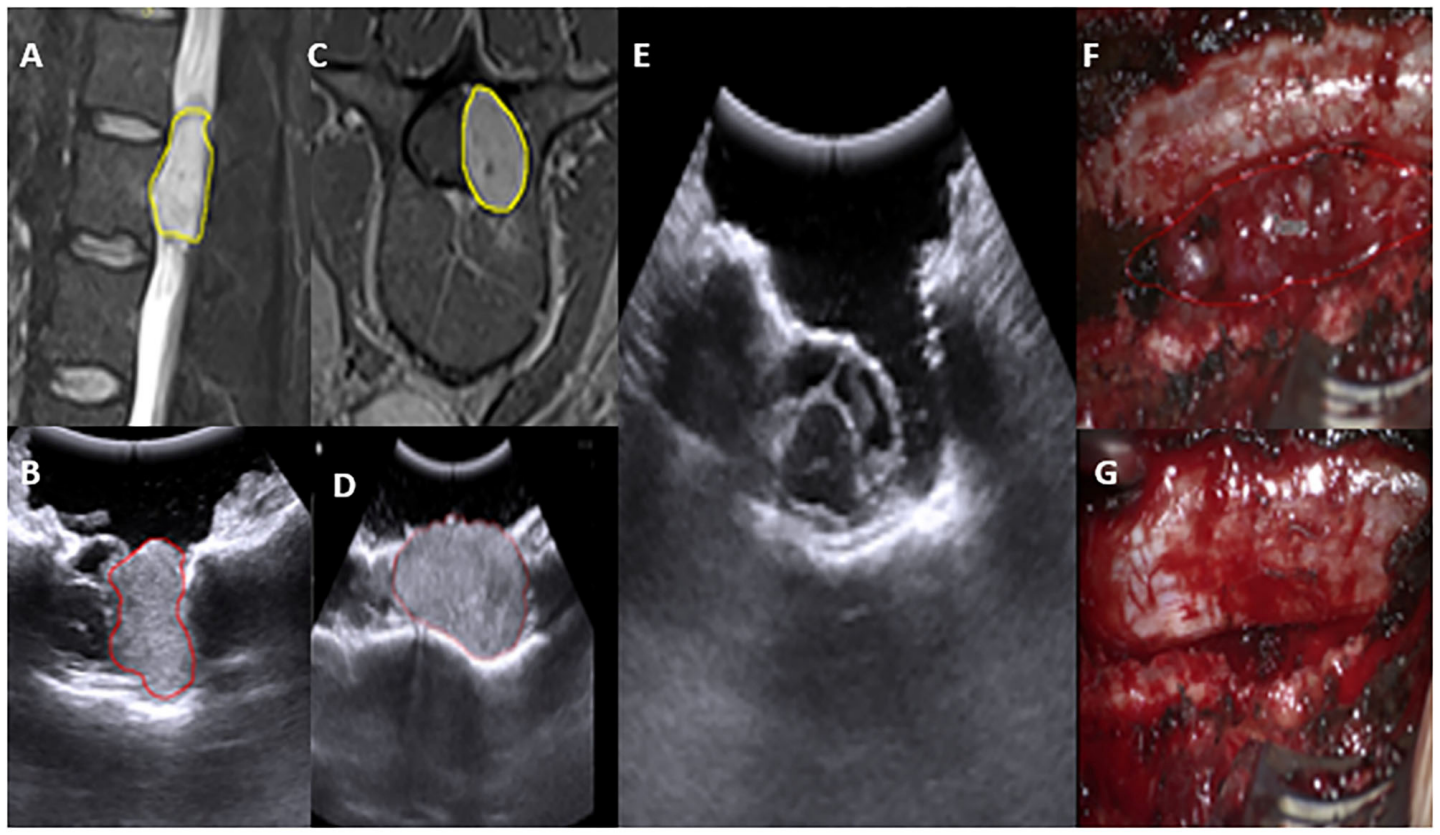
Preoperative MRI T2WI sagittal and axial view depicting an extradural L2–3 lesion **(A,C)** yellow. In concordance, IUS depicting the lesion-location with spinal cord boundary **(B,D)**. Post-operative IUS Clearly shows total resection **(E)**. Intraoperative images, prior and post-resection **(F,G)**.

The iUS is fairly useful for determining the level and localizing the lesion during spine surgery. We have had remarkable success using iUS for both extramedullary and intramedullary lesions, such as neurofibromas, ependymomas, and astrocytomas, in both extramedullary and intramedullary settings. The iUS can be used to safely aid and remove tumor successfully through the surgery, without disturbing the flow of surgery. The iUS made it easier to confirm tumor location and extension, plan myelotomy, and estimate the extent of the lesion (Figure 9).

### Epilepsy Neurosurgery

**Figure 10:**
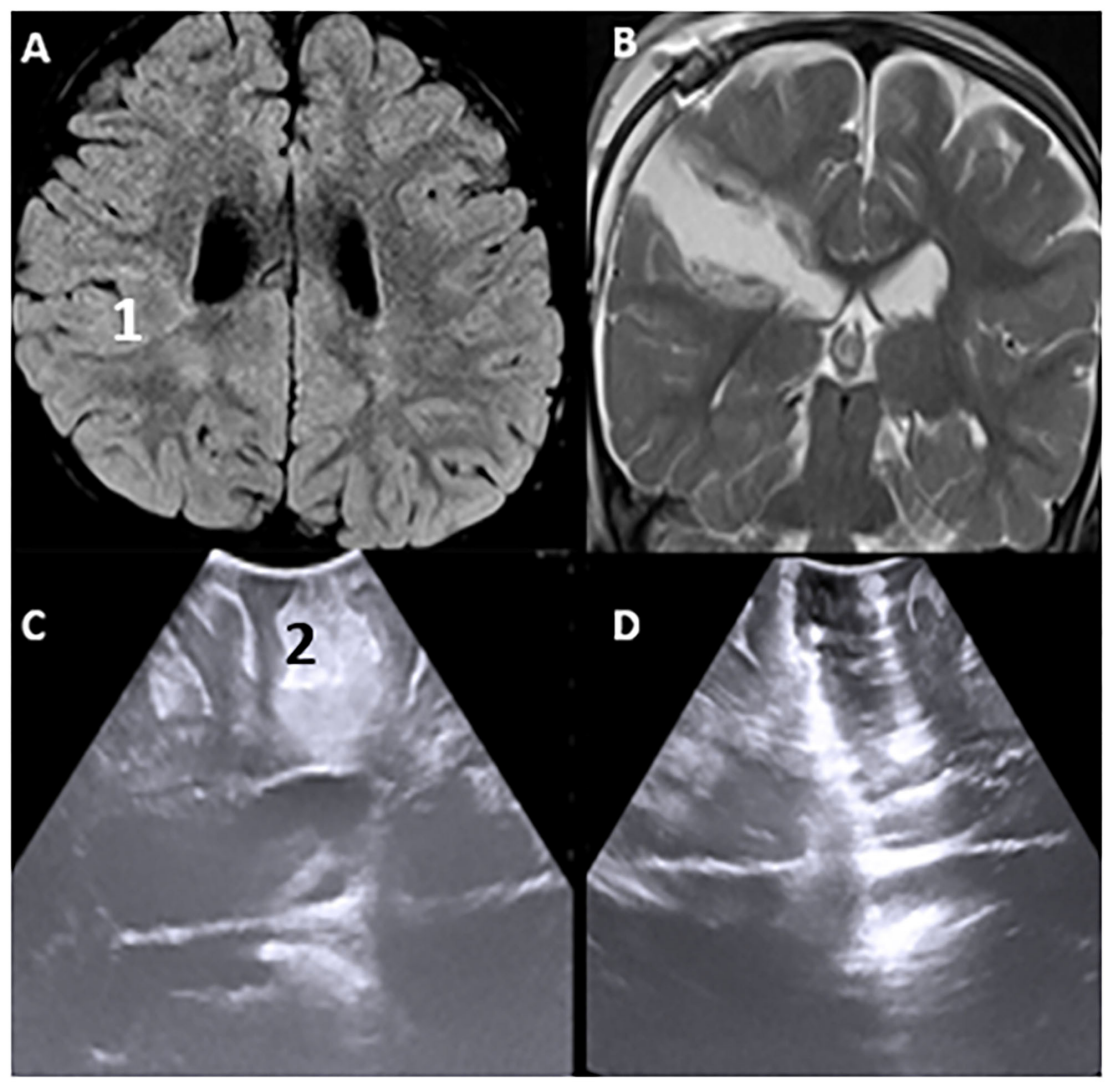
Pre-operative Hyperintence axil MRI FLAIR rt FCD type IIb **(A,1)** in concordance with pre-resection intraoperative hyper echoic cortical and subcortical IUS features **(C,2)**. Post-operative coronal T2WI **(B)** and intraoperative post-resection IUS showing complete resection of the lesion **(D)**.

One of the most common causes of drug intractable epilepsy is focal cortical dysplasia (FCD). In epilepsy surgery, considering that normal and dysplastic brain tissues are often indistinguishable, we have utilized iUS to localize epileptogenic lesions and, thereby, improve surgical outcomes. Cortical characteristics demonstrated perfect concordance between iUS (thickness and hyper-echoic of cortex, subcortical white matter) and MRI T2-weighted/FLAIR images (hyperintense cortical and subcortical changes), especially in Focal cortical type II. The lesion margins on IUS pictures are even clearer than on MRI images. As a result, we believe that iUS is a valuable tool for epilepsy surgery (Figure 10).

## Discussion

As a neuro-navigation modality, iUS navigation in neurosurgery requires a review in terms of utility and user-friendliness, assisting resection extent, and financial burden in terms of purchasing it in neurosurgical centers. The survey conducted suggested there appears to be widespread global availability and familiarity with iUS. Additionally, the general opinion appears to be that the application of iUS during neurosurgery provides relatively quick and accurate information on lesion location and boundaries without negatively affecting intraoperative workflow. The investigators have observed that the introduction of high-resolution iUS during neurosurgery at the study site has provided a relatively convenient and user-friendly modality for intraoperative identification, localization, and characterization of neurosurgical lesions.

Relative to other modes of intraoperative neuronavigation such as MRI and CT-scan, some of the key features and potential advantages of iUS that the investigators have noted include:

- Provision of real-time information on lesion location and extent even in the face of “brain shift”; This maximizes lesion resection while minimizing the negative effects on surrounding healthy tissue.
- Significant reduction in surgical time. A time duration evaluation use of intraoperative MRI was evaluated in a study conducted by Sacino et al. Their study observed that they had an operative time range of 1.5–3 h of additional surgery as a result of the technicalities of performing iMRI. The preparation and transportation of the patient to the MRI cabinet, conducting the MRI, and returning to the operation room were major factors that contributed to additional operative time. Additionally, when back in the theater, re-sterilization, and re-gowning of the patient, nurses, and surgeon compounded into the time factor (7). This was in sharp contrast to our experience in which using iUS did not influence the operational flow. The approximate time taken for each IUS navigation screening session was as follows: (1) prior to dura opening-−1–2 min; (2) after dura opening, lesion localization and delineation are 1–2 min. Control for residual lesion post-resection is 1–2 min.
- Cost-effectiveness about acquisition, running, and maintenance of iUS apparatus – It is important to appreciate that the prohibitively high costs associated with CT- and MRI-based neuronavigation techniques limit their availability and use in LMICs (9).
- Non-invasive nature and associated safety for both patient and neurosurgeon.
- Intraoperative repeatability.

Unfortunately, as is the case with any technique applied in many fields, iUS has some disadvantages, key of which is the steep learning curve. However, the investigators are of the opinion that the rate at which different surgeons will acquire the skill to effectively employ iUS is largely a function of their years of surgical experience (2, 9).

## Conclusion

The application of iUS in neurosurgical practice will continue to evolve in the face of improvements in surgical techniques, as well as new and improved technologies.

## Data Availability Statement

The original contributions presented in the study are included in the article/supplementary material, further inquiries can be directed to the corresponding author.

## Ethics Statement

Written informed consent was obtained from the individual(s) for the publication of any potentially identifiable images or data included in this article.

## Author Contributions

KS, RS, II, and AS: conceptualization. KS, LB, and LM: data curation. KS, AS, and RS: formal analysis. KS: investigation and project administration. RS and LB: resources. LB, RS, II, and LM: software. LB and LM: supervision. KS, RS, and II: validation. KS and RS: roles/writing—original draft and writing—review and editing. All authors contributed to the article and approved the submitted version.

## Conflict of Interest

The authors declare that the research was conducted in the absence of any commercial or financial relationships that could be construed as a potential conflict of interest.

## Publisher's Note

All claims expressed in this article are solely those of the authors and do not necessarily represent those of their affiliated organizations, or those of the publisher, the editors and the reviewers. Any product that may be evaluated in this article, or claim that may be made by its manufacturer, is not guaranteed or endorsed by the publisher.

## Abbreviations

iUS: intraoperative ultrasonography
iMRI: intraoperative magnetic resonance imaging
iCT: intraoperative computed tomography
LMIC: low-&-middle-income countries
MIC: middle-income countries
HIC: high-income countries
CSF: cerebral spinal fluid
FCD: focal cortical dysplasia
AVM: arteriovenous malformation
LGG: low grade glioma
HGG: high-grade gliomas.
